# ﻿Plastome-based subgenus-level phylogenetic backbone of hawthorns: insights into the maternal position and taxonomic synopsis of *Crataegusshandongensis* (Rosaceae, Maleae)

**DOI:** 10.3897/phytokeys.252.136506

**Published:** 2025-02-12

**Authors:** Xiao-Hua Lin, Si-Yu Xie, Guang-Ning Liu, Dai-Kun Ma, Fei-Ran Zou, Wei-Dong Peng, Chao Xu, Bing Liu, Liang Zhao, Bin-Bin Liu

**Affiliations:** 1 College of Life Sciences & Herbarium of Northwest A&F University, Northwest A&F University, Yangling, Shaanxi 712100, China; 2 State Key Laboratory of Plant Diversity and Specialty Crops, Chinese Academy of Sciences, Beijing 100093, China; 3 China National Botanical Garden, Beijing 100093, China; 4 Key Laboratory of National Forestry and Grassland Administration on Plant Ex situ Conservation, China National Botanical Garden (North Garden), Beijing 100093, China; 5 University of Chinese Academy of Sciences, Beijing 100049, China; 6 College of Life Science, Shandong Agricultural University, Taian, Shandong 271018, China

**Keywords:** Chloroplast genome, *Crataegus*, deep genome skimming, lectotype, taxonomy

## Abstract

The recent recognition of the five-subgenera classification within *Crataegus* has prompted discussion about the maternal phylogenetic relationships among these subgenera, with inconsistencies in taxon sampling, marker selection, and inference methods contributing to differing interpretations. In this study, we performed deep genome skimming sequencing and assembled 63 whole plastomes, including 58 from *Crataegus* and five from related genera as the outgroups. We employed multiple phylogenetic inference methods (Maximum Likelihood and Bayesian Inference) to reconstruct an accurate phylogeny. The whole plastome-based, maternally inherited trees consistently supported two major clades within *Crataegus*: one comprising C.subg.Crataegus and C.subg.Brevispinae, the other encompassing the remaining three subgenera. Within the latter clade, C.subg.Sanguineae and C.subg.Americanae formed a sister group, which together were sister to C.subg.Mespilus. Our analysis also revealed a close maternal relationship between *C.shandongensis* and C.pinnatifidavar.major, suggesting the shared maternal ancestry. Furthermore, we updated the description of *C.shandongensis* based on extensive specimen examination and designated the lectotype for this species. This comprehensive taxonomic synopsis, supported by both phylogenomic and morphological analyses, provides a robust foundation for future taxonomic and evolutionary studies of the Shandong hawthorn.

## ﻿Introduction

The hawthorn genus *Crataegus* L. represents a diverse group of deciduous shrubs and small trees within the family Rosaceae, playing a significant ecological role as a source of food and habitat for various pollinators, birds, and mammals. In comparison to the morphologically similar genus *Hesperomeles* Lindl., which is endemic to South America, *Crataegus* is native to temperate regions of North America, Europe, and Asia. These two genera can be easily distinguished by their distinct distributions, although they share several morphological traits, including the presence of thorns, polypyrenous drupes, shoot dimorphism, clusters of small, fragrant white or pink flowers, and bright red or orange fruits (Robertson et al. 1991; Gu and Spongberg 2003; Kalkman 2004; Phipps 2014). The genus *Crataegus* is taxonomically challenging, with the reported number of species varying from 140 to 230 (Phipps et al. 1990; Gu and Spongberg 2003; Phipps 2014). This variability is attributed to several factors, primarily widespread hybridization and polyploidization, especially among North American species (Dickinson 2018; Liston et al. 2021). These processes have resulted in a dynamic genetic landscape, complicating the classification and study of *Crataegus* and making it a compelling subject for botanical and taxonomic research.

Recent molecular phylogenetic and morphological studies have consistently supported the merging of *Crataegus* and *Mespilus* L. (Lo et al. 2007) and proposed a comprehensive subgeneric classification of *Crataegus*, including five subgenera: C.subg.Crataegus, C.subg.Americanae El Gazzar, C.subg.Brevispinae (Beadle) Ufimov & T.A.Dickinson, C.subg.Mespilus (L.) Ufimov & T.A.Dickinson, and C.subg.Sanguineae Ufimov (Ufimov and Dickinson 2020). All the Chinese *Crataegus* species belong to two subgenera: C.subg.Crataegus and C.subg.Sanguineae (Yu and Gu 1974; Gu and Spongberg 2003). Despite these advances, plastid-based phylogenies have yielded conflicting topologies for these five subgenera due to differences in sampling strategies, sequencing regions, and analytical methods (Lo et al. 2007; Zarrei et al. 2014; Liston et al. 2021).

**Figure 1.**
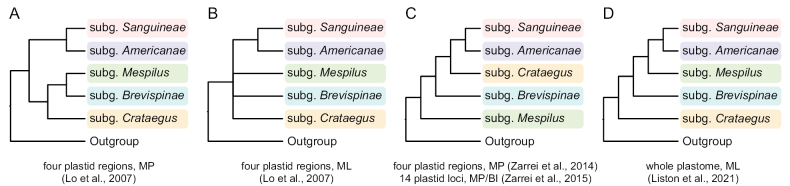
Phylogenetic hypotheses for the five subgenera of *Crataegus* adapted from prior studies: C.subg.Americanae, C.subg.Brevispinae, C.subg.Crataegus, C.subg.Mespilus, and C.subg.Sanguineae. **A, B** Maximum Parsimony (MP) and Maximum Likelihood (ML) trees inferred from four plastid regions (*trn*G-*trn*S, *psb*A-*trn*H, *trn*H-*rpl*2, and *rpl*20-*rps*12), as presented by Lo et al. (2007). **C**MP tree estimated from four plastid regions (*trn*L-F, *trn*G-*trn*S, *rpl*2-*trn*H, and *rpl*20-*rps*12; Zarrei et al. 2014). Additionally, MP tree and Bayesian Inference (BI) tree inferred from 14 plastid loci (*trn*G-*trn*S, *rpl*2-*trn*H, *rpl*20-*rps*12, *trn*L-*trn*F, *atp*B-*rbc*L, *rps*16 intron, *rpl*16 intron, *trn*C-*ycf*6, *acc*D, *rpo*C1, *atp*F-*atp*H, *mat*K, *rbcL*a, and *psb*A-*trn*H; Zarrei et al. 2015). **D**ML tree inferred from whole plastome sequences, as described by Liston et al. (2021).

Lo et al. (2007) sequenced four plastid regions—*trn*G-*trn*S, *psb*A-*trn*H, *trn*H-*rpl*2, and *rpl*20-*rps*12—and analyzed the phylogeny of *Crataegus* using Maximum Parsimony (MP) and Maximum Likelihood (ML) methods. These approaches resulted in differing topologies (Fig. 1A vs. 1B). The MP analysis identified two major clades: one comprising subg. mericanaemericanae and subg. anguineaeanguineae, and the other containing subg. rataegusrataegus, subg. revispinaerevispinae, and subg. espilusespilus. Conversely, the ML analysis, while confirming the sister relationship between subg. mericanaemericanae and subg. anguineaeanguineae, left other inter-subgeneric relationships unresolved. Zarrei et al. (2014) used four plastid sequences (*trn*L-F, *trn*G-*trn*S, *rpl*2-*trn*H, and *rpl*20-*rps*12) with the MP method, producing yet another topology in which subg. espilusespilus was basal, followed by subg. revispinaerevispinae, subg. rataegusrataegus, subg. anguineaeanguineae, and subg. mericanaemericanae (Fig. 1C), and this topology was confirmed by the inclusion of more data (Zarrei et al. 2015). More recently, Liston et al. (2021) analyzed 24 plastomes (14 diploids and ten allotetraploids) and found strong support for subg. rataegusrataegus as the basal clade, followed by a clade containing subg. espilusespilus, subg. mericanaemericanae, and subg. anguineaeanguineae (Fig. 1D). These discrepancies underscore the complexities involved in resolving *Crataegus* phylogeny, which may be attributed to factors such as sampling limitations, methodological differences, and the impact of hybridization and rapid early radiation (Ufimov and Dickinson 2020).

Based on the morphological similarity, Gu and Spongberg (2003) placed *Crataegusshandongensis* F.Z.Li & W.D.Peng in C.subg.Crataegus, and Li and Peng (1986) noted its close morphological relationship with *C.cuneata* Siebold & Zucc. in the protologue. However, the accurate phylogenetic placement of *C.shandongensis* has not been thoroughly estimated since its publication (Li and Peng 1986).

Maternally inherited plastomes have been widely used for phylogenetic inference in angiosperms, particularly within the Rosaceae family. Nikiforova et al. (2013) pioneered the use of whole plastomes to address evolutionary questions in Rosaceae. Advances in sequencing technologies have significantly enhanced the application of plastome data. Zhang et al. (2017) assembled 132 plastomes from across the Rosaceae family, establishing a robust phylogenetic framework that has informed subsequent studies. The PhyloAI team further contributed by assembling plastome and nuclear ribosomal DNA (nrDNA) sequences from deep genome skimming (DGS) data (Liu et al. 2021). Their work has addressed numerous taxonomic and evolutionary questions within Rosaceae, notably in the tribe Maleae (Liu et al. 2019, 2020a, 2020b, 2022, 2023a, 2023b; Jin et al. 2023, 2024a, 2024b; Wang et al. 2024) and *Prunus*-related lineages (Hodel et al. 2021, 2022; Su et al. 2021). This successful application of plastome-based phylogenetic studies enhances our confidence in exploring the maternally subgeneric relationships and phylogenetic placement of *Crataegusshandongensis*.

A comprehensive taxonomic synopsis is crucial for understanding species diversity, supporting conservation efforts, and addressing ecological challenges. It provides a solid foundation for scientific communication and informed decision-making. A refined synopsis of a species should include accurate typification, an updated description and diagnosis, an evaluated conservation status, continuous field observations, and specimen examinations across major herbaria worldwide. In this study, we aim to provide an updated taxonomic synopsis of *Crataegusshandongensis*. In its protologue, Li and Peng (1986) designated the gathering *W.D. Peng 84001*, collected on 15 May 1984, as the type for *C.shandongensis*, specifying that the type was deposited in the herbarium of the Forestry School of Shandong Province, with an “isotype” sent to the China National Herbarium (PE). However, specimens from the Forestry School of Shandong Province herbarium have since been transferred to the herbarium of Shandong Agricultural University (SDAU). A comprehensive examination of the specimens at SDAU by one of our authors, Wei-Dong Peng, found multiple duplicates of *W.D. Peng 84001*, including an inflorescence branch collected on 15 May 1984 and an infructescence branch collected on 2 July 1984. In accordance with Article 40.2 of the International Code of Nomenclature for Algae, Fungi, and Plants (ICN, Turland et al. 2018), when the type is indicated by reference to an entire gathering, or a part thereof, that consists of more than one specimen, those specimens are syntypes. Accordingly, we conclude that the four duplicates of the gathering *W.D. Peng 84001*, collected on 15 May 1984, qualify as syntypes, thereby necessitating the lectotypification of this name.

In this study, we aim to 1) clarify the maternally inherited phylogenetic relationships among *Crataegus* subgenera using multiple inference methods; 2) resolve the phylogenetic placement of *Crataegusshandongensis* through plastome-based phylogenomic analyses in the framework of the hawthorns and 3) provide an updated taxonomic synopsis, including revised typification and morphological description, to facilitate accurate identification and conservation efforts.

## ﻿Materials and methods

### ﻿Taxon sampling

To clarify the maternal relationship among the five subgenera of *Crataegus* and determine the phylogenetic position of *C.shandongensis*, we conducted a comprehensive taxon sampling strategy across the genus. This included all currently recognized subgenera: C.subg.Crataegus, C.subg.Americanae, C.subg.Brevispinae, C.subg.Mespilus, and C.subg.Sanguineae (Ufimov and Dickinson 2020). Specifically, we sampled 16 individuals representing nine species from C.subg.Crataegus. We also sampled 11 individuals from C.subg.Americanae, two from C.subg.Brevispinae, two from C.subg.Mespilus, and 27 from C.subg.Sanguineae. In total, our sampling encompassed 58 individuals from 34 species across all subgenera. To provide a broader phylogenetic context, we included five representative species as the outgroup: *Amelanchiercusickii* Fernald, *Hesperomelesgoudotiana* Killip, *Peraphyllumramosissimum* Nutt., *Malacomelesdenticulata* (Kunth) Decne., and *Malusdomestica* (Suckow) Borkh., resulting in a total of 63 samples (Liu et al. 2020b; Liston et al. 2021). Such extensive sampling significantly enhances the robustness and reliability of our phylogenetic analyses, providing a strong foundation for understanding the evolutionary relationships within *Crataegus*.

For novel data generation, we performed DGS on two newly collected samples, *Crataegusbrachyacantha* Sarg. & Engelm. and *C.shandongensis*. Additionally, we incorporated 36 whole plastome datasets from GenBank at the National Center for Biotechnology Information (NCBI) and 25 plastome datasets from the study of Liston et al. (2021), detailed in Table 1. This integrated dataset provided a robust framework for analyzing maternal genetic relationships within *Crataegus* and for evaluating the phylogenetic placement of *C.shandongensis*.

**Table 1.** The *Crataegus* accessions used in this study are detailed below. Bold rows denote samples sequenced by our PhyloAI team. Accession numbers marked with an asterisk (*) indicate plastomes sequenced specifically for this study. Notably, all 25 plastomes assembled by Liston et al. (2021) had not been submitted to GenBank, and their corresponding accession numbers were absent in this table.

Subgenera	Species	Accession number	Location	Voucher	Publication
subg. mericanaemericanae	*C.calpodendron* (Ehrh.) Medik.	–	Massachusetts, USA	*T.A. Dickinson 2002-07A*	Liston et al. 2021
subg. mericanaemericanae	*C.chrysocarpa* Ashe 1	–	Idaho, USA	*E.Y.Y. Lo EL-122*	Liston et al. 2021
subg. mericanaemericanae	*C.chrysocarpa* 2	–	Washington, USA	*J. Coughlan JC174*	Liston et al. 2021
subg. mericanaemericanae	*C.crus-galli* L.	–	Georgia, USA	*N. Talent NT489*	Liston et al. 2021
subg. mericanaemericanae	*C.macracantha* Koehne 1	–	Washington, USA	*J. Coughlan JC168*	Liston et al. 2021
subg. mericanaemericanae	*C.macracantha* 2	–	Colorado, USA	*N. Talent NT347*	Liston et al. 2021
**subg. mericanaemericanae**	***C.marshallii* Eggl. 1**	**MK920294**	**Minnesota, USA**	***J. Wen 14051* (US)**	**Liu et al. 2019**
subg. mericanaemericanae	*C.marshallii* 2	MK920293	USA	*J.B. Nelson 26961* (US)	Liu et al. 2019
subg. mericanaemericanae	*C.opaca* Hook. & Arn.	–	Louisiana, USA	*T.A. Dickinson 2003-33*	Liston et al. 2021
subg. mericanaemericanae	*C.punctata* Jacq.	–	Ontario, Canada	*M.A. Purich 81*	Liston et al. 2021
subg. mericanaemericanae	*C.triflora* Chapm.	–	Alabama, USA	*T.A. Dickinson 2003-23*	Liston et al. 2021
subg. revispinaerevispinae	*C.brachyacantha* Sarg. & Engelm. 1	–	Louisiana, USA	*C. Reid 5203*	Liston et al. 2021
**subg. revispinaerevispinae**	***C.brachyacantha* 2**	PQ283266*	**Texas, USA**	***J.W. Hardin & R.M. Brown 521* (US03513565)**	**This study**
**subg. rataegusrataegus**	***C.cuneata* Siebold & Zucc. 1**	**OR915925**	**Hubei, China**	***C.M. Zhao et al. EX2548* (PE01857312)**	**Wang et al. 2024**
subg. rataegusrataegus	*C.cuneata* 2	MZ504723	–	–	Unpublished
subg. rataegusrataegus	*C.hupehensis* Sarg. 1	MW201730	Beijing, China	BJLGY-2020-SZ001	Hu et al. 2021a
**subg. rataegusrataegus**	***C.hupehensis* 2**	**OR915900**	**Hubei, China**	***B.B. Liu 2322* (PE02070251)**	**Wang et al. 2024**
subg. rataegusrataegus	*C.laevigata* (Poir.) DC.	OM232780	United Kingdom	–	Liu et al. 2022
subg. rataegusrataegus	*C.monogyna* Jacq. 1	ON641281	Portugal	LISE:96340	Unpublished
subg. rataegusrataegus	*C.monogyna* 2	–	Ontario, Canada	*T.A. Dickinson 2003-79*	Liston et al. 2021
subg. rataegusrataegus	C.pinnatifidavar.major N.E.Br. 1	KY419945	–	Zhang sd148	Zhang et al. 2017
subg. rataegusrataegus	C.pinnatifidavar.major 2	MZ494513	Liaoning, China	JD1H	Hu et al. 2021b
subg. rataegusrataegus	*C.rhipidophylla* Gand.	OM232778	Turkey	–	Liu et al. 2022
subg. rataegusrataegus	*C.scabrifolia* (Franch.) Rehder 1	OP021659	China	SWFU20210783MFY	None
**subg. rataegusrataegus**	***C.scabrifolia* 2**	**OR915927**	**Yunnan, China**	***G.P. Yang 333* (PE01438424)**	**Wang et al. 2024**
**subg. rataegusrataegus**	***C.scabrifolia* 3**	**OR915928**	**Yunnan, China**	***Y.L. Shui et al. 64833* (PE01438422)**	**Wang et al. 2024**
**subg. rataegusrataegus**	***C.shandongensis* F.Z.Li & W.D.Peng**	PQ283267*	**Shandong, China**	***s.coll. s.n.* (PE)**	**This study**
**subg. rataegusrataegus**	***C.songarica* K.Koch 1**	**OR915930**	**Xinjiang, China**	***Z.M. Zhang 222* (PE01153664)**	**Wang et al. 2024**
**subg. rataegusrataegus**	***C.songarica* 2**	**OR915929**	**Xinjiang, China**	***Y.R. Lin 74898* (PE01153668)**	**Wang et al. 2024**
subg. espilusespilus	*C.germanica* (L.) Kuntze 1	MK920295	–	*M.D. Tidestrom 14120* (US)	Liu et al. 2019
subg. espilusespilus	*C.germanica* 2	–	California, USA	*T.A. Dickinson s.n.*	Liston et al. 2021
**subg. anguineaeanguineae**	***C.altaica* (Loudon) Lange 1**	**OR915923**	**Xinjiang, China**	***Z.M. Zhang 263* (PE01153350)**	**Wang et al. 2024**
**subg. anguineaeanguineae**	***C.altaica* 2**	**OR915924**	**Xinjiang, China**	***D.Y. Hong et al. 0136* (PE01153341)**	**Wang et al. 2024**
**subg. anguineaeanguineae**	***C.aurantia* Pojark.**	**OR897856**	**Gansu, China**	***X.G. Sun et al. 2708* (PE01841632)**	**Wang et al. 2024**
subg. anguineaeanguineae	*C.bretschneideri*C.K.Schneid.	MW963339	Beijing, China	BJLGY-2020SZ002	Zheng et al. 2021
subg. anguineaeanguineae	*C.chungtienensis* W.W.Sm. 1	ON032469	Yunnan, China	YUNCM2021051701	Wu et al. 2022
subg. anguineaeanguineae	*C.chungtienensis* 2	KY419947	–	Zhang sd147	Zhang et al. 2017
subg. anguineaeanguineae	*C.douglasii* Lindl. 1	–	Ontario, Canada	*E. Lo EL-11*	Liston et al. 2021
subg. anguineaeanguineae	*C.douglasii* 2	–	Idaho, USA	*E. Lo EL-170*	Liston et al. 2021
subg. anguineaeanguineae	*C.douglasii* 3	–	Oregon, USA	*J. Coughlan JC224*	Liston et al. 2021
**subg. anguineaeanguineae**	***C.kansuensis* E.H.Wilson 1**	**OR915910**	**Shanxi, China**	***D.M. Kong k0229* (PE02070251)**	**Wang et al. 2024**
subg. anguineaeanguineae	*C.kansuensis* 2	MF784433	–	PGP00008	Zhang et al. 2020
subg. anguineaeanguineae	*C.maximowiczii*C.K.Schneid. 1	MZ494512	Liaoning, China	MSZ1H	Hu et al. 2021b
**subg. anguineaeanguineae**	***C.maximowiczii* 2**	**OR915918**	**Jilin, China**	***B.B. Liu et al. 4499* (PE)**	**Wang et al. 2024**
subg. anguineaeanguineae	*C.nigra* Waldst. & Kit.	–	Québec, Canada	*T.A. Dickinson 2318-50*	Liston et al. 2021
subg. anguineaeanguineae	*C.oresbia* W.W.Sm.	ON032470	Yunnan, China	YUNCM2021051702	Wu et al. 2022
subg. anguineaeanguineae	*C.rivularis* Nutt. 1	–	Idaho, USA	*E. Lo EL-199*	Liston et al. 2021
subg. anguineaeanguineae	*C.rivularis* 2	–	Nevada, USA	*T.A. Dickinson 2007-02*	Liston et al. 2021
subg. anguineaeanguineae	*C.rivularis* 3	–	New Mexico, USA	*N. Talent NT357*	Liston et al. 2021
subg. anguineaeanguineae	*C.saligna* Greene 1	–	Utah, USA	*T.A. Dickinson 2004-05*	Liston et al. 2021
subg. anguineaeanguineae	*C.saligna* 2	–	Colorado, USA	*T.A. Dickinson 2001-07*	Liston et al. 2021
**subg. anguineaeanguineae**	***C.sanguinea* Schrad.**	**OR915926**	**Inner Mongolia, China**	***Chifeng Collection Team 2-Z29* (PE02044283)**	**Wang et al. 2024**
subg. anguineaeanguineae	*C.* sp.	OM232779	Canada	–	None
subg. anguineaeanguineae	*C.suksdorfii* (Sarg.) Kruschke 1	–	California, USA	*J. Coughlan JC033*	Liston et al. 2021
subg. anguineaeanguineae	*C.suksdorfii* 2	–	Washington, USA	*P.F. Zika 18485*	Liston et al. 2021
**subg. anguineaeanguineae**	***C.wilsonii* Sarg. 1**	**OR915931**	**Sichuan, China**	***Bashan Collection Team 4974* (PE01872493)**	**Wang et al. 2024**
subg. anguineaeanguineae	*C.wilsonii* 2	–	Massachusetts, USA	*T.A. Dickinson s.n.*	Liston et al. 2021
**subg. anguineaeanguineae**	***C.wilsonii* 3**	**OR915932**	**Sichuan, China**	***Bashan Collection Team 5683* (PE01872495)**	**Wang et al. 2024**
**outgroup**	***Amelanchiercusickii* Fernald**	**MN068257**	**Washington, USA**	***J.W. Thompson 14588* (US1889072)**	**Wang et al. 2024**
outgroup	*Hesperomelesgoudotiana* Killip	MN068271	Colombia	*J. Cuatrecasas 5035*	Unpublished
**outgroup**	***Peraphyllumramosissimum* Nutt.**	**KY420011**	**Nevada, USA**	***B. Maguire & A.H. Holmbren 25288* (US03513221)**	**Wang et al. 2024**
**outgroup**	***Malacomelesdenticulata* (Kunth) Decne.**	**MN068267**	**Mexico**	***A. Ventura 3359* (US03513083)**	**Wang et al. 2024**
outgroup	*Malusdomestica* (Suckow) Borkh.	–	–	–	Liston et al. 2021

### ﻿DNA extraction, library preparation, and DGS sequencing

We extracted whole genomic DNA from herbarium specimens of *Crataegusbrachyacantha* and *C.shandongensis* using a modified CTAB (mCTAB) protocol, based on the methods described by Doyle and Doyle (1987) and adapted by Li et al. (2013). This extraction was performed at the Plant DNA and Molecular Identification Platform (PDMIP) at the Institute of Botany, Chinese Academy of Sciences (IBCAS). Skilled technicians ensured the successful extraction of high-quality DNA from the often degraded material of herbarium specimens.

DNA libraries were then prepared using the NEBNext^®^ Ultra^™^ II DNA Library Prep Kit, involving careful quality control to ensure the libraries were suitable for high-throughput sequencing. This preparation included fragmentation, end-repair, and adapter ligation of the DNA to create libraries compatible with the sequencing platform. The DGS sequencing was conducted on the BGISEQ-500 platform at Frasergen in Wuhan, China. Each sample generated approximately 20 gigabases (Gb) of raw data with 150 base pair (bp) paired-end reads, providing a comprehensive whole genomic DNA dataset for in-depth genomic analysis.

The raw sequencing data have been archived in the NCBI Sequence Read Archive (SRA) under BioProject accession number PRJNA1155316. The corresponding voucher specimens are preserved in the PE and the United States National Herbarium (US), ensuring that both the genomic data and physical specimens are accessible for future research and validation.

### ﻿Raw data cleaning and quality control

The raw sequencing data were initially processed to enhance quality by trimming low-quality reads and removing adapter sequences using Trimmomatic v. 0.39 (Bolger et al. 2014). This tool applied various filtering steps, including removing sequences below a specified quality threshold (LEADING:3, TRAILING:3, SLIDINGWINDOW:4:15, MINLEN:36) and eliminating adapter contaminants that could interfere with subsequent analyses. This trimming process retained only high-quality, reliable reads, forming a robust dataset for further genomic work.

Following trimming, we assessed the quality of the clean reads using FastQC v. 0.12.1 (Andrews 2010, available at https://www.bioinformatics.babraham.ac.uk/projects/fastqc/). FastQC provided detailed reports on various quality metrics, including read length distribution, GC content, sequence duplication levels, and potential overrepresented sequences. This quality assessment was crucial for validating the effectiveness of the trimming process and ensuring that the data met the necessary standards for assembly.

We proceeded with chloroplast genome assembly only after confirming the integrity and quality of the clean reads. The careful preprocessing and quality control ensured that our assembly was based on the most accurate and reliable sequence data, thereby enhancing the robustness and reliability of the subsequent genomic analyses.

### ﻿Plastome assembly and annotation

Given the variability in sequencing coverage across samples, we employed the Successive Approach combining Reference-based and *de novo* assembly (SARD approach, Liu et al. 2023b), a method that performs well even for the low-coverage data. This assembly approach has been successfully applied to a range of angiosperm families, including Amaryllidaceae (Lou et al. 2022a), Magnoliaceae (Liu et al. 2020e; Wang et al. 2020), Rosaceae (Liu et al. 2019, 2020b, 2022, 2023a, 2023b; Jin et al. 2023, 2024b), and Vitaceae (Liu et al. 2021).

We utilized two distinct programs for automatic plastome assembly, each employing different algorithms: mapping-and-*de novo* assembly with GetOrganelle v. 1.7.7.0 (Jin et al. 2020) and seed-and-extend assembly with NOVOPlasty v. 3.6 (Dierckxsens et al. 2017). Initially, GetOrganelle v. 1.7.7.0 was used with default parameters to assemble plastomes, successfully generating a high-quality circular plastome for *Crataegusshandongensis*. For the non-circular *C.brachyacantha* sample, NOVOPlasty was applied with parameters set to “Genome Range = 120000-200000”, “K-mer = 31”, “Read Length = 150”, and “Insert size = 300” using the *rbc*L sequence of *C.brachyacantha* (accession number: KC251218) as the seed and the plastome of *C.pinnatifida* Bunge (accession number: MN102356) as the reference. This method did not produce a circular plastome either. Consequently, we employed the SARD approach for assembly. We aligned plastome-related reads from the raw reads of *C.brachyacantha* to the reference plastome of *C.maximowiczii*C.K.Schneid. (accession number: NC065485) using Geneious Prime v. 2023.2 (Kearse et al. 2012) to generate a consensus sequence. Concurrently, we performed *de novo* assembly with SPAdes v. 3.13.1, applying error correction and K-mer lengths of 21, 33, 55, and 77 (Bankevich et al. 2012). Finally, we mapped all contigs assembled from SPAdes, GetOrganelle, and NOVOPlasty to the consensus sequence, resulting in a high-quality, complete circular plastome.

The plastomes were then annotated using GeSeq (Tillich et al. 2017). All assembled plastomes from this study have been submitted to GenBank, with accession numbers provided in Table 1.

### ﻿Data matrix generation and phylogenetic inference

In this study, we utilized the whole plastome dataset for phylogenetic inference. We applied concatenation-based method for estimation. We retained only one of the inverted repeats (IRs) from each plastome of *Crataegus* and outgroups, including all 63 plastomes. The alignment of these plastomes was performed with MAFFT v. 7.520 (Nakamura et al. 2018) using the “--auto” parameter and then refined with trimAl v. 1.4.1 (Capella-Gutiérrez et al. 2009) to exclude poorly aligned regions, applying parameters “-gt 0.8 -st 0.001”.

For concatenation-based phylogenetic inference, we utilized various programs and algorithms for accurate estimation, including ML and Bayesian Inference (BI). Optimal evolutionary models were determined using PartitionFinder2 (Lanfear et al. 2014, 2017; Stamatakis 2014) with parameters for “linked branch lengths”, “Akaike Information Criterion model (AICc)”, and “greedy search” approach (Lanfear et al. 2012). We inferred ML trees using IQ-TREE2 v. 2.2.2.7 (Minh et al. 2020), with 1,000 ultrafast bootstrap support assessments (Minh et al. 2013) and the SH-aLRT test (Anisimova and Gascuel 2006) for evaluating tree topology reliability. Additionally, we used RAxML v. 8.2.13 (Stamatakis 2014) for ML tree inference, with parameters “-f a -p 12345 -x 12345” and 200 rapid bootstrap replicates. For BI analysis, MrBayes 3.2.7a (Ronquist et al. 2012) was employed, with Markov Chain Monte Carlo (MCMC) analysis set to run for 10 million generations, sampling trees every 1,000 generations and discarding the initial 25% of samples.

### ﻿Morphological analysis

To update the taxonomic synopsis of *Crataegusshandongensis*, we examined specimens housed at several related herbaria, including Lushan Botanical Garden (LBG), PE, Qufu Normal University (QFNU), and SDAU. Notably, we focused on duplicates of the type collection (*W.D. Peng 84001*) at the herbarium SDAU. Key diagnostic traits, including leaf morphology, inflorescence structure, and fruit characteristics, were documented and measured using a stereo microscope.

### ﻿Data resources

The data underpinning the analysis reported in this paper are deposited in the Dryad Data Repository at https://doi.org/10.5061/dryad.z612jm6nc.

## ﻿Results and discussion

### ﻿DGS sequencing and data generation

Our PhyloAI team generated 17 DGS raw datasets for the genus *Crataegus*, including newly sequenced data for *C.brachyacantha* and *C.shandongensis* (Table 1). The DGS sequencing produced 19.89 Gb of data for *C.brachyacantha* and 16.46 Gb for *C.shandongensis*, with coverage depths of 24.16× and 19.99×, respectively, based on the estimated genome size of 823.41 Mb for C.pinnatifidavar.major N.E.Br. (Zhang et al. 2022). This high coverage ensures reliable plastome assembly and accurate data for phylogenetic analyses.

**Figure 2.**
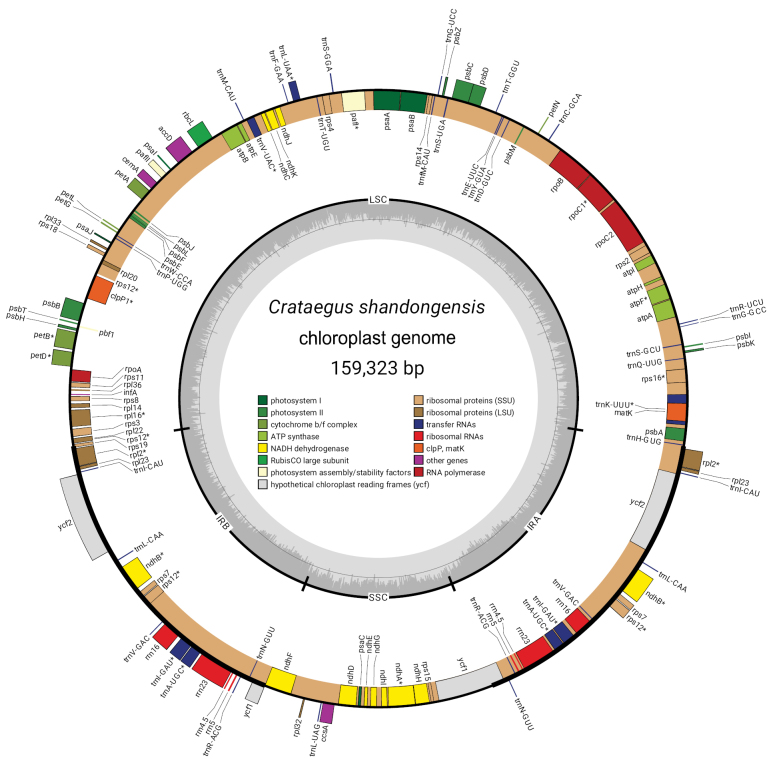
Gene map of the *Crataegusshandongensis* chloroplast genome, with genes inside the circle transcribed clockwise and those outside transcribed counterclockwise. The dark gray inner circle indicates GC content, while the light gray outer circle denotes AT content. Different gene types are represented in various colors. LSC, large single copy; SSC, small single copy; IRA, inverted repeat region A; IRB, inverted repeat region B.

We assembled complete circular plastomes for two species (Table 1), marking the first plastome assemblies for *Crataegusbrachyacantha* in the monotypic subgenus C.subg.Brevispinae and for the China-endemic *C.shandongensis* in C.subg.Crataegus. Each plastome in this study featured two IRs that were complementary, separated by a small single copy (SSC) and a large single copy (LSC) region (Fig. 2). Overall, the plastomes encoded 133 genes: 88 protein-coding genes, 37 tRNA genes, and eight rRNA genes (Fig. 2). No significant rearrangements or gene losses were found in all the representative species of all five subgenera. These plastomes provide crucial insights into species relationships within *Crataegus* and are essential for accurately resolving the phylogenetic relationships among the five currently recognized subgenera. To enhance the precision of our phylogenetic analyses, we performed extensive data cleaning, resulting in whole plastome dataset, with alignment lengths of 130,986 bp. These high-quality plastome datasets are invaluable resources for future research in the phylogenetics, taxonomy, and evolutionary biology of *Crataegus*, underscoring the utility of DGS in studying non-model organisms and advancing our understanding of their biodiversity.

### ﻿Well-resolved maternal phylogenetic relationship among five subgenera in *Crataegus*

**Figure 3.**
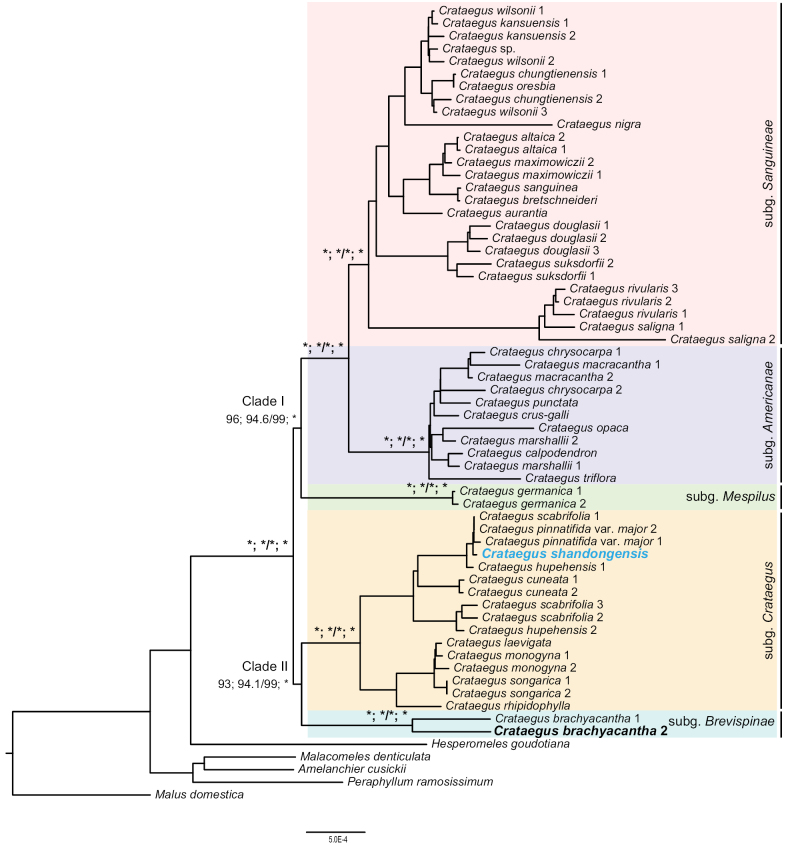
Phylogenetic tree of *Crataegus* estimated using the Maximum Likelihood (ML) algorithm with RAxML based on the whole plastome dataset. Support values for key nodes are shown next to the branches: Bootstrap (BS) from RAxML, SH-aLRT support and ultrafast bootstrap from IQ-TREE2, and posterior probability (PP) from Bayesian analysis based on whole plastome dataset. Asterisks (*) indicate full support (100; 100/100; 1). Detailed support values refer to Suppl. materials 1–3.

In this study, we collected whole plastome data covering all five currently recognized subgenera of *Crataegus* to accurately estimate their maternal relationships. We constructed three trees based on the whole plastome dataset using two phylogenetic inference methods (ML and BI, Fig. 3, Suppl. materials 1–3). All whole plastome-based trees revealed consistent topologies, dividing the five *Crataegus* subgenera into two well-supported clades, designated as Clade I and Clade II (Fig. 3, Suppl. materials 1–3). Clade I comprises three subgenera, and C.subg.Mespilus is sister to a combined clade including C.subg.Americanae and C.subg.Sanguineae (bootstrap support (BS) = 96, SH-aLRT support/ultrafast bootstrap support (SH-aLRT/UFBoot) = 94.6/99, posterior probabilities (PP) = 1; Suppl. materials 1–3). Crataegussubg.Americanae and C.subg.Sanguineae were consistently resolved as sister groups in all three trees with robust support (BS = 100, SH-aLRT/UFBoot = 100/100, PP = 1; Fig. 3, Suppl. materials 1–3). Clade II includes two subgenera, C.subg.Crataegus and C.subg.Brevispinae (BS = 93, SH-aLRT/UFBoot = 94.1/99, PP = 1; Fig. 3; Suppl. materials 1–3).

In Crataegussubg.Sanguineae, the plastome with GenBank accession number OM232779, initially identified as *C.mollis* (Torr. & A.Gray) Scheele, was assembled from whole genomic data (SRA accession no. SRR3130998) provided by the Harvard University Arnold Arboretum. Unexpectedly, this sample clusters with C.subg.Sanguineae rather than with C.subg.Americanae, which contradicts previous morphological evidence, such as differences in mature fruit color (Phipps 2014). This discrepancy raises the possibility of misidentification or DNA contamination, possibly due to issues during field collection or sample labeling. The GenBank record only lists “USA” as the locality and lacks voucher specimen information, which limits our ability to verify its identity. We recommend that future submissions to GenBank include detailed locality and voucher information to improve verification and reproducibility. Liu et al. (2022) utilized this plastome as an outgroup in an apple study but did not detect its anomalous placement due to limited taxon sampling. The broader sampling in our current study, which included increased taxonomic representation and better resolution, has brought this inconsistency to light. To confirm the identity and phylogenetic placement of OM232779, further investigation is required. Until such data are available, we recommend interpreting the phylogenetic position of this sample with caution, particularly for any downstream analyses or conclusions that may rely on this placement.

### ﻿Phylogenetic position and taxonomic synopsis of *Crataegusshandongensis*

All three phylogenetic trees consistently supported a close maternal relationship among *Crataegusshandongensis*, C.pinnatifidavar.major, and *C.scabrifolia* (Franch.) Rehder (Fig. 3; Suppl. materials 1–3). Notably, *C.scabrifolia* appeared in two separate clades (Fig. 3; Suppl. materials 1–3), suggesting multiple maternal sources for this species. *Crataegusshandongensis* is endemic to Shandong, *C.scabrifolia* is found in South and Southwest China (including Chongqing, Guangxi, Guizhou, Sichuan, and Yunnan), and C.pinnatifidavar.major is distributed across South, Southeast, East, and North China, as well as the Korean Peninsula and the Far East of Russia. Given their distributions, it is plausible to infer that *C.shandongensis* is more closely related to C.pinnatifidavar.major maternally.

**Figure 4.**
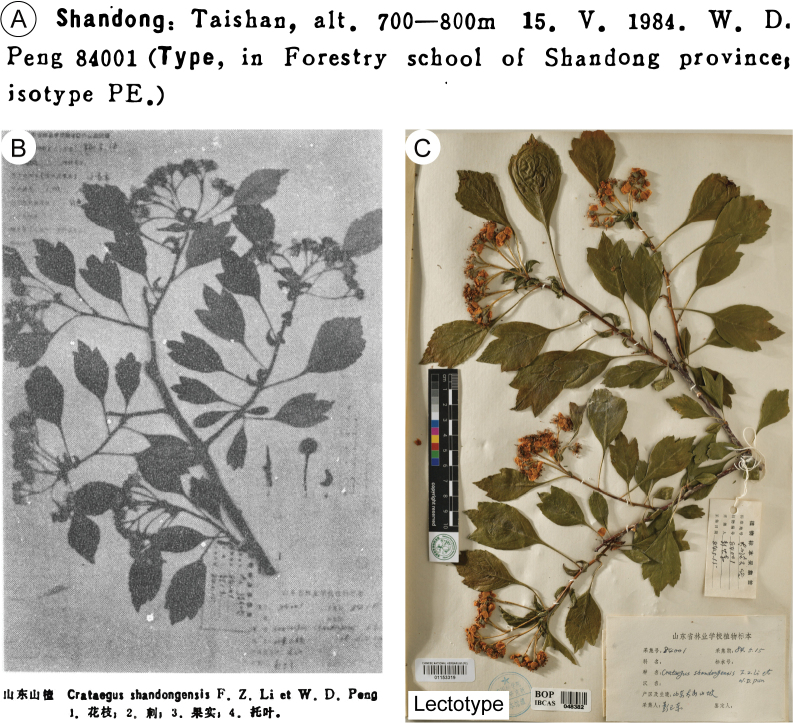
Type specimens of *Crataegusshandongensis*. **A** typification information of *Crataegusshandongensis* in the protologue (Li and Peng 1986) **B** duplicate of *W.D. Peng 84001* shown in the protologue (Li and Peng 1986) **C** lectotype. Collection number: *W.D. Peng 84001*; Collection date: 15 May 1984; Collector: W.D. Peng; Collection location: Taishan, Shandong, China.

Li and Peng (1986) labeled two gatherings, the flowering branch collected on 15 May 1984 and the fruiting branch collected on 2 July 1984, to be *W.D. Peng 84001*. This practice of collecting both flowering and fruiting specimens from the same individual was common in the 20^th^ century, especially in collections by E.H. Wilson in China (Liu et al. 2020c, 2020d; Lou et al. 2022b; Wang et al. 2024). Early plant collectors often deemed it essential to obtain specimens from the same individual in different stages of development. Many collectors attached permanent tags to the plants to track these collections and sometimes retained the original collector’s number for subsequent collections, noting the different collection dates and/or collectors. This practice has significantly impacted the accurate typification of related species. For instance, in the case of *Photiniaschneideriana* Rehder & E.H.Wilson, Wilson combined branches collected in May and October 1907 onto one sheet and designated it as the type for *P.schneideriana*. This action contradicted Article 8.2 of ICN (Turland et al. 2018), a case that has been thoroughly discussed (Liu and Hong 2017; Lou et al. 2022b). Fortunately, Li and Peng (1986) clearly indicated the type information (Fig. 4A), specifying the gathering collected on 15 May 1984 as type, with type deposited in the Forestry School of Shandong Province herbarium and isotype sent to PE. It should be noted that the sense of isotype in the protologue of Li and Peng (1986) was syntype (Art. 40.2 in Turland et al. 2018). Thus, the presence of specimens with the same collector number but different collection dates does not affect the valid publication of *C.shandongensis*.

**Figure 5.**
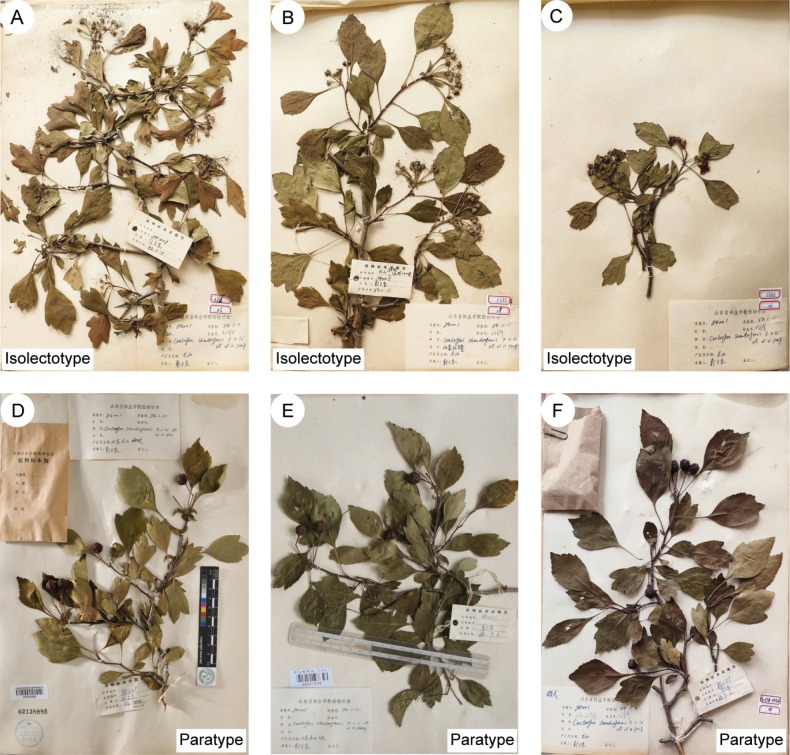
Type specimens of *Crataegusshandongensis*. **A** isolectotype. Collection number: *W.D. Peng 84001*; Specimen accession number: 2365; Collection date: 15 May 1984; Collector: W.D. Peng; Collection location: Taishan, Shandong **B** isolectotype. Collection number: *W.D. Peng 84001*; Specimen accession number: 2364; Collection date: 15 May 1984; Collector: W.D. Peng; Collection location: Taishan, Shandong **C** isolectotype. Collection number: *W.D. Peng 84001*; Specimen accession number: 2368; Collection date: 15 May 1984; Collector: W.D. Peng; Collection location: Taishan, Shandong **D** paratype (PE [barcode 01153320]). Collection number: *W.D. Peng 84001*; Collection date: 2 July 1984; Collector: W.D. Peng; Collection location: Taishan, Shandong **E** paratype (LBG [barcode 00021556]). Collection number: *W.D. Peng 84001*; Collection date: 2 July 1984; Collector: W.D. Peng; Collection location: Taishan, Shandong. **F** Paratype (SDAU!). Collection number: *W.D. Peng 84001*; Collection date: 2 July 1984; Collector: W.D. Peng; Collection location: Taishan, Shandong.

The authors provided an image of a specimen that includes both an inflorescence branch and fruit (Fig. 4B). However, we were unable to locate this particular duplicate mentioned by the original authors. We suspect that this specimen may have been lost or destroyed during the transfer from the herbarium of the Forestry School of Shandong Province to the SDAU herbarium. Fortunately, we identified three additional duplicates in the herbarium SDAU and one in PE, and all these four duplicates are syntypes (Figs 4C, 5A–C). According to Article 9.12 of ICN (Turland et al. 2018), syntypes and isosyntypes share equal priority for lectotypification. Consequently, we selected a well-preserved duplicate in PE (barcode 01153319) as the lectotype (Fig. 4C), as the three duplicates in SDAU have been severely damaged by insects, particularly impacting the petals of the flowers (Fig. 5A–C).

#### ﻿ Crataegus shandongensis

Taxon classificationPlantaeRosalesRosaceae

F.Z.Li & W.D.Peng, Bull. Bot. Res., Harbin 6: 149. 1986.

9E1EB34C-80C7-5950-A097-57413C27BDAE

##### Type.

China. Shandong, Taian, Taishan, alt. 700–800 m, 15 May 1984, *W.D. Peng 84001* (***lectotype***, **designated here**: PE [barcode 01153319!, Fig. 4C]; isolectotypes: SDAU!, Fig. 5A–C).

##### Description.

Shrubs 1–2 m tall, thorns 1–1.2 cm long or absent; branchlets initially pubescent and then glabrous, older branches gray-brown, sparsely lenticellate, glabrous. Stipules herbaceous, falcate, margin glandular-serrate. Leaves simple, deciduous; petioles 1.5–4 cm long, narrowly winged, glabrous; blades 4–8 cm long, 2–4 cm wide, obovate or long-elliptic; base cuneate, apex acuminate; usually 3-lobed, rarely 5-lobed or unlobed; margins irregularly double-serrate above the middle part; venation craspedodromous; glabrous adaxially, except for the loosely hairy midrib, pinnate veins slightly impressed above, prominent below, veins entering the tip of the serrations. Inflorescences corymb, 7–18 flowered, 4 cm long, 8 cm wide; pedicels and peduncles white-pubescent; bracts linear-lanceolate 2–3 mm long, margin glandular-serrate, caducous; hypanthium externally white-pubescent; small flower 2 cm in diameter; petals suborbicular, white, about 5 mm long, very shortly clawed; sepals triangular, apex caudate-acuminate, 4–6 mm long, nearly as long as the calyx tube, white-pubescent adaxially; stamens 20; styles 5, base white-villous. Fruits globose, 10–15 mm in diameter, red, glabrous; sepals persistent, reflexed; nutlets 5, flat on both sides, grooved on the back.

##### Distribution.

China (Shandong: Mount Tai).

##### Conservation status.

*Crataegusshandongensis* is currently known from a single extant population situated along the slope of Mount Tai. Despite thorough field observations over the past 38 years by one of the authors (Wei-Dong Peng), no additional populations have been found. Although Mount Tai has been under continuous protection for over 5,000 years due to its profound cultural significance, which has helped to preserve the natural habitat to some extent, the species remains restricted to this limited area. According to the IUCN Red List Criteria (IUCN 2022), specifically criteria B1ab(iii)+2ab(iii), *C.Shandongensis* meets the thresholds for Critically Endangered (CR) status. These criteria pertain to the restricted geographic range (criterion B1) and the limited area of occupancy (criterion B2), coupled with an ongoing decline in habitat quality (subcriterion ab(iii)). The restricted distribution and the absence of additional populations justify the classification of *C.shandongensis* as CR.

##### Additional specimens examined

**(paratypes).** China. Shandong, Mount Tai (Taishan), altitude 700–800 m, 2 July 1984, *W.D. Peng 84001* (PE [barcode 01153320!, Fig. 5D], LBG [barcode 00021556!, Fig. 5E], SDAU! [Fig. 5F]).

## ﻿Conclusions

In summary, our plastome-based phylogenomic analyses of all five recognized subgenera have provided a detailed clarification of their phylogenetic relationships. We successfully resolved the maternal lineage of *Crataegusshandongensis*, confirming its distinct position within the genus and deepening our understanding of its phylogenetic context. Furthermore, all Shandong hawthorn-related specimen examinations throughout the Chinese herbaria enabled us to develop a comprehensive taxonomic synopsis for *C.shandongensis*, including its typification and an updated species description.

While the maternally inherited plastomes are valuable for elucidating maternal lineages, they have limitations in addressing complex evolutionary processes such as hybridization and polyploidization (Guo et al. 2022). As we transition into the phylogenomic era—marked by the utilization of hundreds or thousands of single-copy nuclear genes (SCNs)—substantial progress is anticipated in plant systematics (Liu et al. 2021). Research integrating both maternally inherited plastid DNA and biparentally inherited SCNs has already highlighted the importance of detecting conflicts between these compartments, offering new insights into hybridization and polyploidy (Lo et al. 2007; Liston et al. 2021; Jin et al. 2023). Additionally, Doyle (2022) provided a critical review for accurately inferring species trees using coalescent methods, emphasizing the integration of diverse genomic resources, including SCN genes, plastid protein-coding genes, and mitochondrial genes. This study provides a crucial foundation for the phylogenomic era, offering a robust framework to facilitate and advance future research endeavors.

## Supplementary Material

XML Treatment for Crataegus shandongensis

## ﻿Additional information

### Author ORCIDs

Xiao-Hua Lin https://orcid.org/0009-0001-2528-8384

Si-Yu Xie https://orcid.org/0000-0002-0227-0982

Guang-Ning Liu https://orcid.org/0009-0009-0765-0392

Dai-Kun Ma https://orcid.org/0009-0005-5523-508X

Fei-Ran Zou https://orcid.org/0009-0008-7817-826X

Wei-Dong Peng https://orcid.org/0009-0001-0176-8367

Chao Xu https://orcid.org/0000-0002-9678-4772

Bing Liu https://orcid.org/0000-0002-6086-253X

Liang Zhao https://orcid.org/0000-0003-3846-505X

Bin-Bin Liu https://orcid.org/0000-0002-0297-7531

### Data availability

All of the data that support the findings of this study are available in the main text or Supplementary Information.

## Supplementary materials

Supplementary material 1 Whole plastome-based phylogenetic backbone of *Crataegus* estimated through Maximum Likelihood analysis using RAxML Data type pdf Explanation note Whole plastome-based phylogenetic backbone of *Crataegus* estimated through Maximum Likelihood analysis using RAxML. The numbers displayed above each branch represent the bootstrap support value. https://binary.pensoft.net/file/1248844This dataset is made available under the Open Database License (http://opendatacommons.org/licenses/odbl/1.0/). The Open Database License (ODbL) is a license agreement intended to allow users to freely share, modify, and use this Dataset while maintaining this same freedom for others, provided that the original source and author(s) are credited.Xiao-Hua Lin, Si-Yu Xie, Guang-Ning Liu, Dai-Kun Ma, Fei-Ran Zou, Wei-Dong Peng, Chao Xu, Bing Liu, Liang Zhao, Bin-Bin Liu

Supplementary material 2 Whole plastome-based phylogenetic backbone of *Crataegus* estimated through Maximum Likelihood analysis using IQ-TREE2 Data type pdf Explanation note Whole plastome-based phylogenetic backbone of *Crataegus* estimated through Maximum Likelihood analysis using IQ-TREE2. The numbers displayed above each branch represent the SH-aLRT support and ultrafast bootstrap support values, respectively. https://binary.pensoft.net/file/1248845This dataset is made available under the Open Database License (http://opendatacommons.org/licenses/odbl/1.0/). The Open Database License (ODbL) is a license agreement intended to allow users to freely share, modify, and use this Dataset while maintaining this same freedom for others, provided that the original source and author(s) are credited.Xiao-Hua Lin, Si-Yu Xie, Guang-Ning Liu, Dai-Kun Ma, Fei-Ran Zou, Wei-Dong Peng, Chao Xu, Bing Liu, Liang Zhao, Bin-Bin Liu

Supplementary material 3 Whole plastome-based phylogenetic backbone of *Crataegus* estimated through Bayesian Inference analysis using MrBayes Data type pdf Explanation note Whole plastome-based phylogenetic backbone of *Crataegus* estimated through Bayesian Inference analysis using MrBayes. The numbers displayed above each branch represent the posterior probability. https://binary.pensoft.net/file/1248846This dataset is made available under the Open Database License (http://opendatacommons.org/licenses/odbl/1.0/). The Open Database License (ODbL) is a license agreement intended to allow users to freely share, modify, and use this Dataset while maintaining this same freedom for others, provided that the original source and author(s) are credited.Xiao-Hua Lin, Si-Yu Xie, Guang-Ning Liu, Dai-Kun Ma, Fei-Ran Zou, Wei-Dong Peng, Chao Xu, Bing Liu, Liang Zhao, Bin-Bin Liu
