# Supplementary figures and images for: ﻿Plastome-based subgenus-level phylogenetic backbone of hawthorns: insights into the maternal position and taxonomic synopsis of Crataegusshandongensis (Rosaceae, Maleae)
Source: PhytoKeys. 2025 Feb 12;252:87–108. doi: 10.3897/phytokeys.252.136506 (PMC11840429; doi:10.3897/phytokeys.252.136506)

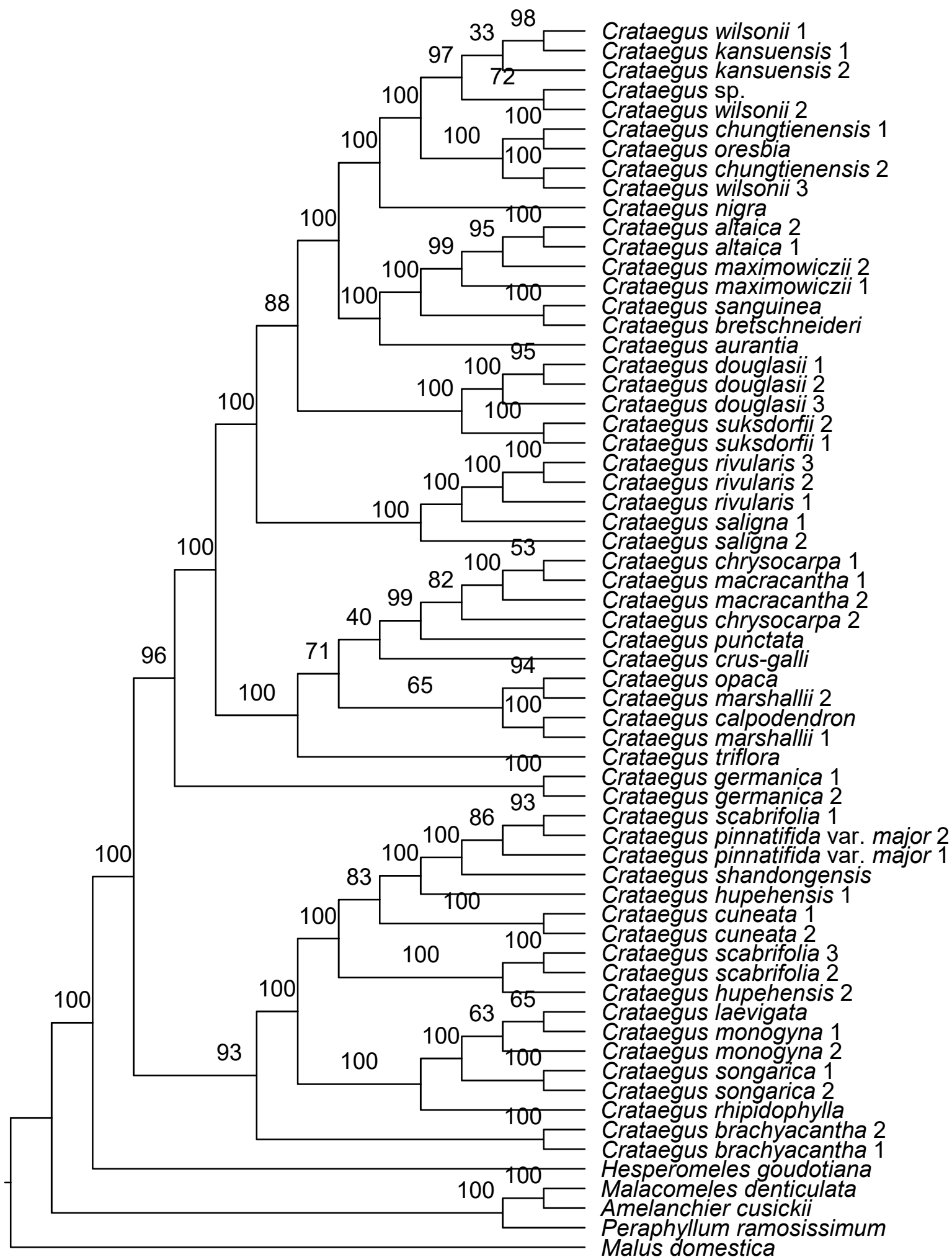

Supplement: Supplementary material 1 — Whole plastome-based phylogenetic backbone of Crataegus estimated through Maximum Likelihood analysis using RAxML [file phytokeys-252-087_article-136506__-s001.pdf]

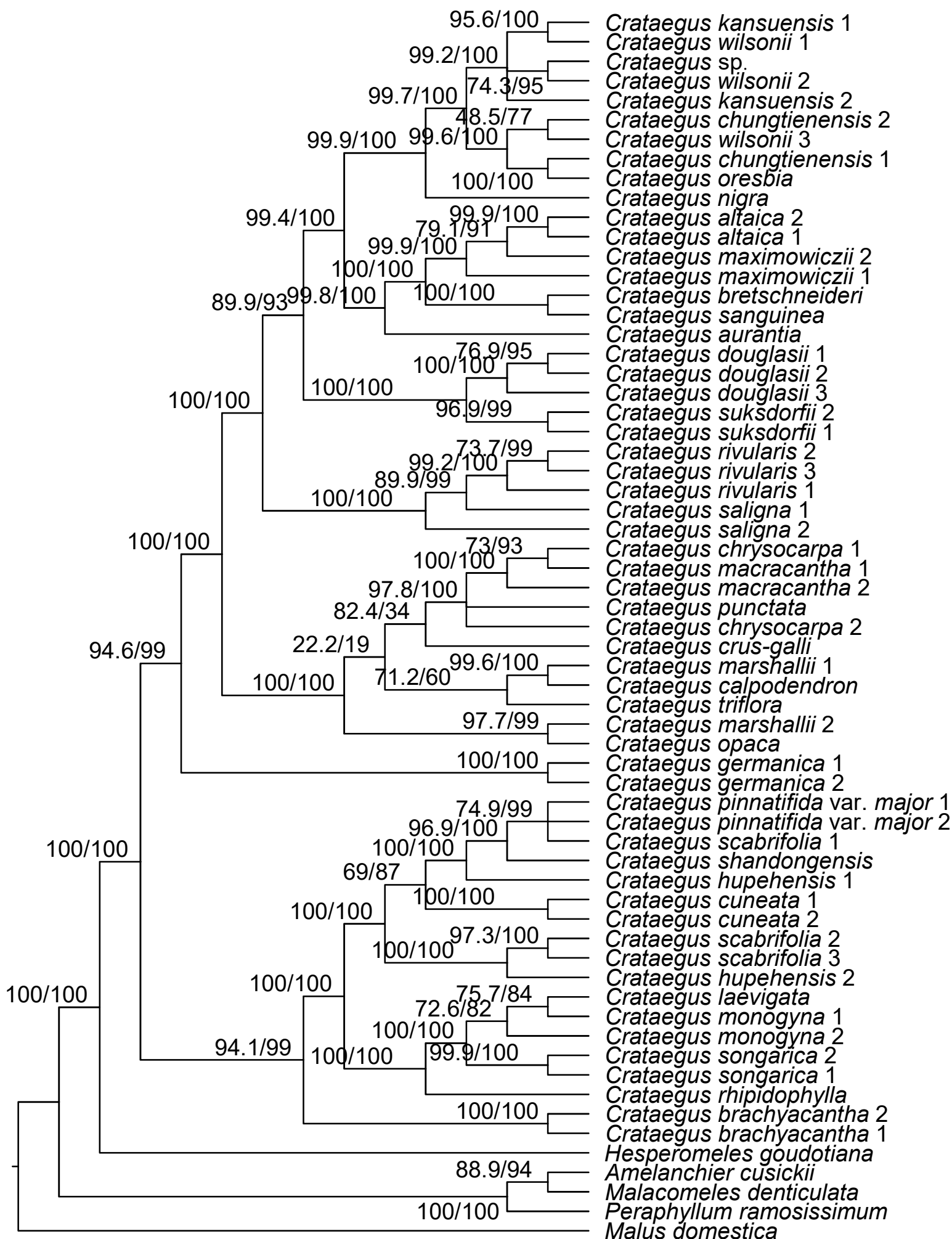

Supplement: Supplementary material 2 — Whole plastome-based phylogenetic backbone of Crataegus estimated through Maximum Likelihood analysis using IQ-TREE2 [file phytokeys-252-087_article-136506__-s002.pdf]

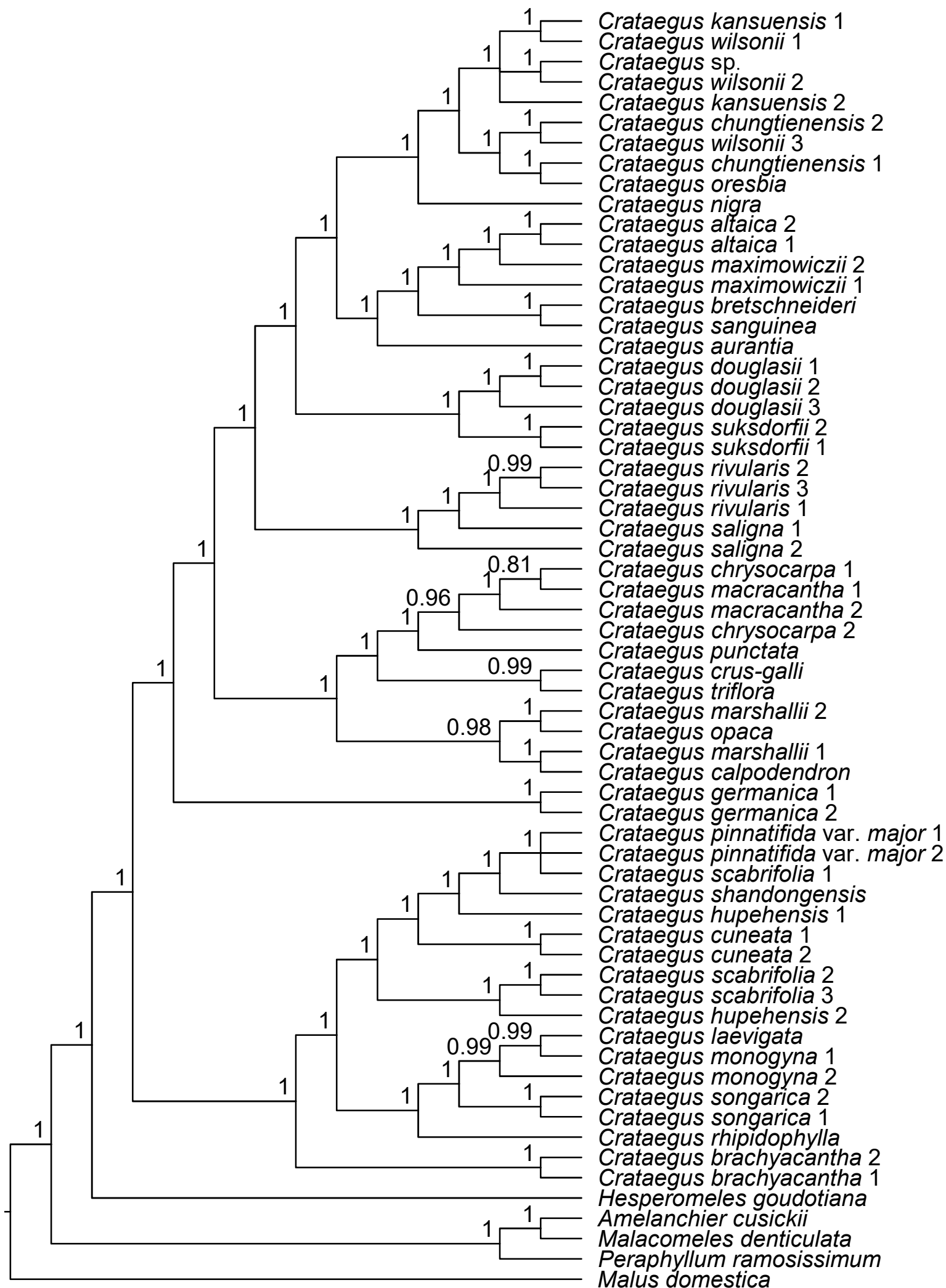

Supplement: Supplementary material 3 — Whole plastome-based phylogenetic backbone of Crataegus estimated through Bayesian Inference analysis using MrBayes [file phytokeys-252-087_article-136506__-s003.pdf]
